# Management of C2 fractures following multiple classifications, a narrative review

**DOI:** 10.1016/j.bas.2024.102928

**Published:** 2024-08-15

**Authors:** Michael McDermott, Guisela Quinteros, Federico Landriel, Chase Stastny, Daniel Raskin, Guillermo Ricciardi, Andrei Fernandes Joaquim, Charles Carazzo, Amna Hussein, Jahangir Asghar, Alfredo Guiroy

**Affiliations:** a Duly Health and Care, 808 Rickert Dr, Naperville, IL, 60540, United States; b Orthopedics Department, Av Vitacura 5951, Vitacura, Región Metropolitana, Chile; c Neurocirugía, Hospital Italiano de Buenos Aires, Tte. Gral. Juan Domingo Perón 4190, C1199, Cdad. Autónoma de Buenos Aires, Argentina; d Kettering Health Dayton, 405 W Grand Ave, Dayton, OH, 45405, United States; e School of Medical Sciences of the Santa Casa de Sao Paulo. R. Jaguaribe, 155 - Vila Buarque, São Paulo, SP, 01224-001, Brazil; f Sanatorio Güemes. Av. Córdoba 3933, C1188AAF, Cdad. Autónoma de Buenos Aires, Argentina; g University of Campinas. Cidade Universitária Zeferino Vaz - Barão Geraldo, Campinas, SP, 13083-970, Brazil; h University of Passo Fundo. BR 285 Km 292,7 | Campus I, Bairro São José - São José, Passo Fundo, RS, 99052-900, Brazil; i Department of Neurosurgery, University of Arizona College of Medicine. 475 N 5th St, Phoenix, AZ 85004, United States; j Elite Spine Health and Wellness. 499 NW 70th Ave STE 200, Plantation, FL, 33317, United States

**Keywords:** Upper cervical fractures, Cervical fractures, Hangman fracture, Odontoid fracture, axis fracture

## Abstract

**Introduction:**

Classifications are helpful for surgeons as they can be a resource for decision-making, often providing the individual indicators that may deem a case necessary for surgery. However, when there are multiple classifications, the decision-making might be compromised. That is the case with C2 fractures. For this reason, this study was designed to review the different classifications of axis fractures.

**Research question:**

What are the most commonly used classifications for C2 fractures, and how do these classifications compare in terms of clinical utility?

**Methods:**

A systematic literature review following the Preferred Reporting Items for Systematic Reviews and Meta-Analysis (PRISMA) Guidelines was performed. Three different Pub-med searches (https://pubmed.ncbi.nlm.nih.gov/) were done to isolate the most common C2 fracture classifications of odontoid process fractures, the posterior element of the axis and axis body fractures.

**Results:**

The search isolated 530 papers. Applying the inclusion and exclusion criteria yielded seven papers on axis body fractures, six on odontoid fractures, and ten on “hangman's fractures.” Most of the classifications proposed are modified versions of the classic ones: Benzel's for body fractures, Anderson and D'Alonzo's for odontoid fractures, and Effendi's for “hangman's fractures.” The proposal by AO Spine of a different classification seems promising and had good early results of interobserver and intraobserver agreement.

**Discussion and conclusion:**

Currently, no classification is universally accepted or widely used. The emergence of the AO Spine Upper Cervical Injury Classification system seems promising as it encompasses radiological and clinical elements.

## Highlights

• There are multiple classifications for C2 fractures.
• No classification is universally accepted or widely used.
• The AO Spine Upper Cervical Injury Classification system seems promising.

## 1 Introduction

Injury to the cervical spine has been reported in 3.7% of all traumas, with spinal instability present in 41.9% of injuries (Burke and Harris, 1989). Early diagnosis and proper management are crucial as these injuries can be severely debilitating, causing neurological deficits, cervical deformities, and leading to chronic pain (Burke and Harris, 1989). Different investigators have proposed classification systems centered around traumatic cervical spine injuries to optimize patient outcomes. Classifications are helpful for surgeons as they can be a resource for decision-making, often providing the individual indicators that may deem a case necessary for surgery. While traditionally helpful, a surplus of different classifications can be overwhelming and increase the difficulty of isolating the best treatment approach for individual cases.

This conundrum is particularly present regarding axis fractures (C2 Vertebra). The axis's unique motion, position, and anatomical characteristics make it prone to many fractures and dislocations, setting it apart from other vertebrae (Hadley et al., 1985). These elements, combined with the complex anatomic relationships of the surrounding structures, have posed a significant challenge in developing a unified and comprehensive classification system. Currently, C2 fractures are divided into three clinically relevant categories: odontoid fractures, posterior elements of the axis fractures (also known as Hangman's fracture or traumatic spondylolisthesis), and axis body fractures (Hadley et al., 1985). No unified classification for all axis injuries has been universally accepted due to the many injury patterns that can affect this vertebra. Thus, this study aims to review the most historically used axis classifications and highlight the differences to clarify the individual characteristics of C2 fractures.

## 2 Material and methods

**Fig. 1:**
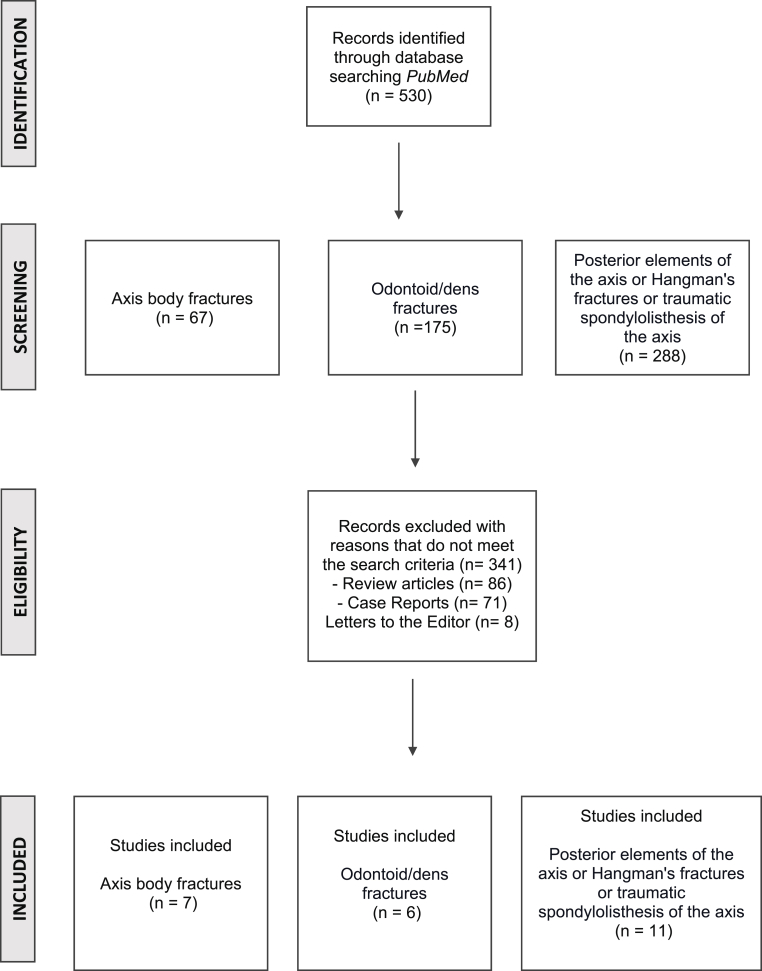
Systematic Review Flowchart including inclusion and exclusion criteria.

A systematic literature review following the Preferred Reporting Items for Systematic Reviews and Meta-Analysis (PRISMA) Guidelines was performed (Page et al., 2021). (See Fig. 1)

Three different Pub-med searches (https://pubmed.ncbi.nlm.nih.gov/) were done to isolate the most common C2 fracture classifications of odontoid process fractures, the posterior element of the axis and axis body fractures. The results of these searches were individually tracked and recorded. Included in this study were manuscripts that described classifications for traumatic injuries affecting C2 and case series that proposed a morphological classification for these traumatic injuries. Articles excluded from the study included biomechanical studies, non-traumatic C2 fractures (tumors and infections), letters to the editor, commentary, opinion articles, studies without a transparent methodology and case reports. The exact search terms for each of the three searches are explicitly described in the appendix. Two authors independently reviewed each manuscript for inclusion in this study (MM, DR). For any discrepancy, the senior author independently reviewed the article (AG) before a group discussion, including the study factoring to a majority vote.

## 3 Results

The systematic review yielded 530 articles, of which 24 were included. Individually, the search for axis body fractures yielded 67 relevant papers, and seven manuscripts were finally selected to be included in this study. See Fig. 1.

The individual characteristics of each study were isolated and summarized in Table 1. The search for odontoid/dens fractures yielded 175 relevant articles, of which six qualified for inclusion in this study. Individual details are summarized in Table 2. Lastly, the review of fracture of posterior elements of the axis or Hangman's fractures or traumatic spondylolisthesis of the axis yielded 288 total studies, of which 11 qualified for inclusion in this analysis (Table 3).

**Table 1:** Summary of the studies selected for axis body fractures.

Title	Author and year	N	Study design	Results	Classification Reliability
**1- Fractures of the C-2 vertebral body**	**Benzel et al., 1994**	15	Case series	3-years retrospective review. Description of mechanism of injury and proposal of new classification.	Not reported
**2- Classification and Treatment of Axis Body Fractures**	**Fujimura et al., 1996**	31	Case series	Case report and proposal of a new classification The author shows results and preferences of treatment. Nonoperative treatment as initial therapy Atlanto-axial fusion occurs when severe malalignment of the atlantoaxial joint is recognized. In sagittal fractures, eight patients had sequelae for A-A Osteoarthritis • Philadelphia brace caused nonunion significantly in fracture displacement >3 mm compared to Minerva brace/Halo vest. • Surgery or rigid Minerva brace/Halovest should be considered for Type 2 transverse fractures of the C2 body with fracture displacement >3 mm.	Not reported
**3- Nonoperative Management of Vertical C2 Body Fractures**	**German et al., 2005**	21	Retrospective review	8-year retrospective review. Treatment: In most patients, nonoperative treatment seems appropriate.	Not reported
**4- Management of Typical and Atypical Hangman's Fractures**	**Al-Mahfoudh, 2015**	41	Case series	Single-center study; 6-year retrospective review. Case report and proposal of new classification. Only 3% atypical hangman's fractures required surgical fixation.	Not reported
**5- A Novel Classification for Atypical Hangman Fractures and Its Application**	**Li et al., 2017**	62	Case series	Single-center study. Case report and proposal of new classification complementary to Levine–Edwards. 27 underwent surgical treatment. 19 patients underwent nonoperative treatment	Not reported
**6- Proposal of New Radiological Classification and Treatment Strategy for Transverse Fractures of the C2 Axis Body**	**Kim et al., 2021**	49	Retrospective review	17 years F.U. Multicenter study Case report and proposal of new classification Treatment: • Majority of transverse fractures can be treated conservatively.	Correlation coefficients for intra-observer and inter-observer reliabilities of classification were 0.723 and 0.598 (both, P < 0.001).
**7- Update on Upper Cervical Injury Classifications: The New AO Upper Cervical Spine Classification System**	**Vaccaro, 2022**	–	Expert Consensus	Created by AO Spine Knowledge Forum Trauma based on published literature and Knowledge Forum Trauma discussions. Considers 3 anatomic categories based on the condyle/vertebra involved and its caudal joint: (I) occipital condyle and craniocervical junction, (II) C1 ring and C1−C2 joint, and (III) C2 and C2−C3 joint. Injury patterns according to modes of mechanical failure include: type A isolated bony injury, type B-ligamentous and tension band injury, and type C-displacement or translation through disk or joint.	For fracture site (I, II, and III), “almost perfect” interobserver reliability (κ = 0.862/0.884 first/second assessment) as well as intraobserver reproducibility for both residents (κ = 0.830−0.999) and senior spine surgeons (κ = 0.861−0.999) was achieved. The interobserver reliability for reported for subtype (A, B, and C) was “substantial” (κ = 0.660/0.603 first/second assessment) and intraobserver reproducibility ranged from “substantial” to “almost perfect” (κ = 0.691−0.920) for residents and “almost perfect” (κ = 0.841−0.983) for senior spine surgeons.

**Table 2:** Summary of the studies selected for odontoid fractures.

Title	Author and year	N	Study design	Results	Classification Reliability
**1- Fractures of the odontoid process of the axis**	**Anderson D'Alonzo, 1974**	49	Case series	Type I: Avulsion fractures of the tip Type II: Fractures of the body of the odontoid process Type III: Fractures of the base of the odontoid process Widely used and accepted	Not reported
**2- Acute axis fractures: a review of 229 cases**	**Hadley, 1988**	229	Case series	Type IIA: Large posterior fragment comminution Modification in D'Alonzo's classification	Not reported
**3-**	**Pedersen, 1994**	1	Retrospective review	Type I: tip of the dens, one alar ligament and transverse ligament intact Type II: IIA: junction of body and dens, with comminution IIB: junction of body and dens, without comminution IIC: above accessory ligament Type III: involves the body of axis	Not reported
**4- Proposal of a modified, treatment-oriented classification of odontoid fractures**	**Grauer, 2004**	52	Case series	Type I: Above inferior aspect or C1 anterior arch Type II: Between type I and III IIA: Non-displaced IIB: Anterior superior to posterior inferior, or displaced transverse IIC: Anterior inferior to posterior superior or comminuted Type III: Includes the superior articular facets Modification in D'Alonzo's classification, prosed treatments for each one of the classifications	The overall kappa value was 0.48, indicating moderate agreement. The lowest level of agreement was observed for Type IIA and Type IIC fractures.
**5- Update on Upper Cervical Injury Classifications: The New AO Upper Cervical Spine Classification System**	**Vaccaro, 2022**		Expert Consensus	Type III: C2 and C3 joint: - Type IIIA fractures include one or more C2 dens, body, pedicle, or posterior arch fractures without associated ligamentous or discal injury. M1 modifier is used to describe injuries at high risk of nonunion with nonoperative treatment, such as a fracture through the waist of the odontoid with significant angulation or displacement. M2 modifier describes injuries with a significant potential for instability. M2 modifier describes injuries with a significant potential for instability. M3 modifier is used to incorporate patient-specific factors affecting treatment. M4 modifier describes a vascular injury or abnormality of the vertebral artery	Excelent reliability. - For fracture site (I, II, and III) = “almost perfect” interobserver reliability (κ = 0.862/0.884 first/second assessment). - For fracture subtype (A, B, and C) = “substantial” interobserver reliability (κ = 0.660/0.603 first/second assessment).
**6- Reevaluation of a classification system: stable and unstable odontoid fractures in geriatric patients-a radiological outcome measurement**	**De Luca, 2022**	89	Retrospective review	Type II D'Alonzo should be classified as Stable and Unstable. Surgery indication for the unstable fractures. Proposed for elderly patients.	Not reported

**Table 3:** Summary of the studies selected for Hangman's fractures.

Title	Author and year	N	Study design	Results	Classification Reliability
**1- Fractures of the ring of the axis. A classification based on the analysis of 131 cases.**	**Effendi et al., 1981**	131	Case series	Type I: Isolated hairline fracture in axis ring Type II: Displacement of anterior fragment and abnormal disk below axis Type III: Displacement of anterior fragment and locked facet C2-C3 Widely known and accepted	Not reported
**2- Traumatic spondylolisthesis of the axis**	**Francis et al., 1981**	123	Case series	Type I: Displacement <3.5 mm and angulation <11° Type II: Displacement <3.5 mm and angulation >11° Type III: Displacement >3.5 mm or <0.5 vertebral width and angulation >11° Type IV: Displacement >3.5 mm or >0.5 vertebral width and angulation >11° Type V: any disk disruption	Not reported
**3- The management of traumatic spondylolisthesis of the axis**	**Levine, Edwards 1985**	52	Case series	Type I: Non-displaced fracture Type II: Significant angulation and translation Type IIa: Severe angulation without translation Type III: Severe angulation and displacement with facet dislocation	Not reported
**4- Atypical Hangman's fractures**	**Starr, Eismont 1993**	63	Case series	Atypical Hangman's fracture: posterior axis body fracture with unilateral or bilateral continuity to the posterior cortex or pedicle	Not reported
**5- The traumatic spondylolisthesis of the axis**	**Josten, 1999**		Narrative review	They distinguished injuries affecting the anterior longitudinal ligament (ALL) as a distinct category. Type I: Intact ALL and an intact disk. Type 2: Intact ALL and a ruptured disk. Type 3: ALL and disk ruptured. Type 4: Posterior dislocated and fixated facet joint.	Not reported
**6- Hangman's fracture: a clinical review based on surgical treatment of 15 cases**	**Goel, 2021**	15	Retrospective analysis	Type 1: No atlantoaxial or C2-C3 instability Type 2:C2-C3 instability, no atlantoaxial instability Type 3: Presence of atlantoaxial instability and no C2-C3 instability Type 4: Both atlantoaxial instability and C2-C3 instability	Not reported
**7- Update on Upper Cervical Injury Classifications: The New AO Upper Cervical Spine Classification System**	**Vaccaro, 2022**		Expert Consensus	Type III: C2 and C3 joint: - Type IIIA fractures include one or more fractures of the C2 dens, body, pedicle, or posterior arch without associated ligamentous or discal injury. M1 modifier is used to describe injuries at high risk of nonunion with nonoperative treatment, such as a fracture through the waist of the odontoid with significant angulation or displacement. M2 modifier describes injuries with a significant potential for instability. M2 modifier describes injuries with a significant potential for instability. M3 modifier is used to incorporate patient-specific factors affecting treatment. M4 modifier describes a vascular injury or abnormality of the vertebral artery	Excelent reliability. - For fracture site (I, II, and III) = “almost perfect” interobserver reliability (κ = 0.862/0.884 first/second assessment). - For fracture subtype (A, B, and C) = “substantial” interobserver reliability (κ = 0.660/0.603 first/second assessment).

## 4 Discussion

The information currently available on the various subtypes of C2 fracture is heterogeneous and varies among different classifications. This review aims to summarize the current classifications to offer surgeons a path to identifying the correct treatment for their patients. Due to the significant variation in fracture patterns documented in the literature, we have elected to separate this article into subtypes of C2 fractures to remain consistent with previously published manuscripts. Our subtype analysis follows the groups reported by Robinson et al., in 2017 when they approached C2 fractures by categorizing them into three general subtypes: axis body fractures (atypical C2 fractures), odontoid fractures, and Hangman's fractures (Robinson et al., 2017).

### 4.1 Axis body fractures

Fractures of the body of C2 are not uncommon but can be challenging to classify as there is a considerable variation in the specific fracture patterns observed. Due to the challenges these fractures present, Benzel et al. proposed a classification based on a combination of the radiological analysis and mechanism of injury. In this classification, type 1 had a horizontally oriented fracture, type 2 had a vertically oriented fracture, and type 3 was described as a horizontal and forward-facing fracture (Benzel et al., 1994). Shortly after this classification was described, Fujimura et al. adjusted this classification into four specific groups: avulsion, transverse, burst, and sagittal; however, this classification has limited use in clinical practice and is not widely used among the spinal community (Fujimura et al., 1996). In 2005, German et al. examined 21 patients with sagittal and coronal fractures of the axis body. They reported that classifying them into different groups was clinically irrelevant as both fracture patterns were treated non-surgically with external orthoses, regardless of the fracture orientation (German et al., 2005). In 2015 Al-Mahfoudh et al. expanded on Benzel's classification by incorporating oblique fracture patterns and lesions that extended unilaterally to the pedicles. They proposed a modified classification where type 1 still represents a coronally oriented fracture, type 2a was defined as an oblique lesion associated with unilateral pars fracture, and type 2b was an oblique trajectory associated with contralateral lamina fractures (Al- et al., 2016). In 2017, Li et al. emphasized that a proper atypical hangman fracture (AHF) classification should include the four Levine-Edwards classification types (Levine and Edwards, 1985; Li et al., 2017). Li further noted that C2 fractures with a coronal orientation cannot be compared to atypical hangman's fractures since the vertebral canal remains intact and proposed a new classification, A1: oblique fracture involving the posterior cortex of C2 on one side associated with a contralateral pars fracture; A2: oblique fracture through one side of the C2 body associated with a contralateral lamina fracture; B1: bilateral oblique fractures through the posterior cortex of C2; B2: bilateral fractures, but one is oblique and the other is vertical (Li et al., 2017). In 2021, a Korean group examined transverse axis body fracture patterns. They developed a classification based on whether the fracture trajectories involve the C2 superior articular facet (SAF) and lateral cortex (LC) on coronal CT scans (Kim et al., 2021). Their resulting classification was divided into three groups: type 1 - fracture trajectories that involve the C2 superior articular facet on both sides; type 2 - fracture trajectories that involve the SAF on one side and the lateral cortex on the other side; type 3 - fracture trajectories that involve the LC on both sides.

Interestingly, there was no difference in the surgical indications. Still, this classification provides indications for using different external orthosis devices: Philadelphia collar was more appropriate for type 1, while type 2 required a rigid Minerva Brace (Kim et al., 2021). Overall, type 2 had less favorable results for the study than type 1, with higher fracture displacement observed (Kim et al., 2021).

In 2022, AO Spine developed a new upper-cervical fracture classification system (AO Spine UCCS). Three anatomically distinct segments were described: 1) the occipital condyle and craniocervical junction; 2) the C1 ring and C1–C2 joint; and 3) the C2 body, odontoid process and C2–C3 joints. Injury types are also classified for each segment, with type A being predominantly bony injuries and typically stable injury patterns. Type B injuries involve a bony and/or ligamentous injury with no vertebral body translation respective to the caudal and cephalad vertebrae. They may be stable or unstable and usually require additional imaging with dynamic radiographs or magnetic resonance imaging (MRI) to determine if operative management is indicated. Type C injuries involve either a ligamentous or bony injury that results in the translation of the proximal and distal parts of the injured spinal column in any plane. These injuries are unstable and frequently require operative stabilization (Vaccaro et al., 2022a). Axis body fractures fall into types 3A-C in this classification. Maeda et al. examined the reliability of the new AO Spine classification on 32 patients. While they found some intra and interobserver variability, they concluded it was a reproducible and safe method for recommending treatment (Maeda et al., 2020). Urrutia et al. also set out to evaluate this classification in 84 patients. They observed moderate inter-observer reliability, and the authors attributed this to the classification's attempt to incorporate multiple fractures into one system (Urrutia et al., 2023). Vaccaro et al. sought to validate this classification globally by examining its use by 275 AO members; their study showed that the classification demonstrated a high interobserver and intraobserver reliability (Vaccaro et al., 2022b).

### 4.2 Odontoid fractures

**Fig. 2:**
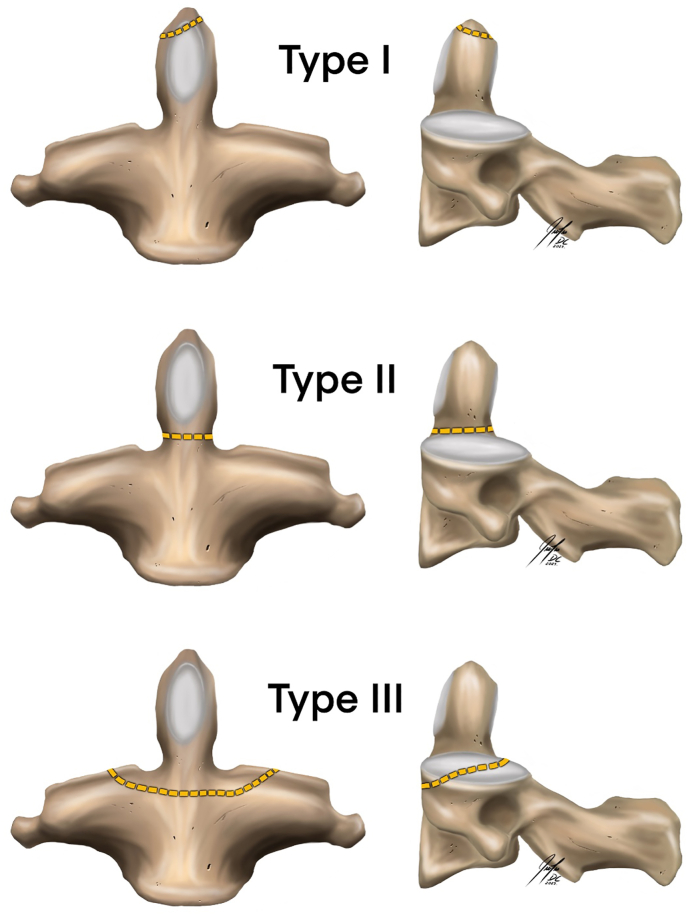
Schematic drawing of the Anderson and D'Alonso classification with its three types of fracture patterns.

The most common type of C2 fracture occurs through the odontoid process. The most widely accepted classification for this type of fracture is the one that Anderson and D'Alonzo established in 1974 (Anderson and D'Alonzo, 1974) (See Fig. 2)

This classification is based on the location of the fracture in the odontoid process, as type I is an avulsion of the tip, type II includes fractures of the neck of the process, and type III involves fractures of the base of the odontoid process. Several studies have argued that this classification may be too simple, given the potential diversity of fracture patterns within each location, particularly for those grouped in type 2. This concern has led to several proposals to modify this classification, mostly centered around providing subgroups to type 2 fractures. Hadley et al. were the first to suggest the addition of a subgroup to the original classification in 1988, when they proposed adding a type IIA group characterized by fragmentation at the dens fracture site (Hadley et al., 1989). In 1994, Pedersen et al. highlighted the need for a more precise classification, proposing that type I should be defined as the tip of the dens, one alar ligament, and transverse ligament intact. Type IIA is a fracture at the junction of the body and dens, with comminution, IIB is the same fracture without comminution, and IIC is a fracture above the accessory ligament. Type III is a fracture that involves the body of the axis (Pedersen and Kostuik, 1994). In 2005, Grauer et al. proposed a modification of the Anderson and D'Alonzo classification based on the treatments for each injury pattern. In this modification, type I are fractures above the inferior aspect or C1 anterior arch, and type III involves superior articular facets. Type II is the fracture that lies between type I and III, and is subclassified based on fracture pattern. Type IIA are non-displaced fractures, type IIB is anterior superior to posterior inferior or displaced transverse fractures, and type IIC is anterior inferior to posterior superior or comminuted fractures (Grauer et al., 2005). In 2022, Deluca et al. investigated expanding the operative indications in these classifications by studying odontoid fractures in the elderly. Deluca emphasized that not all patients with type II fractures required surgical treatment and suggested the need to subdivide type II D'Alonzo as stable and unstable, suggesting treating stable type II fractures non-operatively with semi-rigid immobilixation (Deluca et al., 2022).

### 4.3 Hangman's fractures

**Fig. 3:**
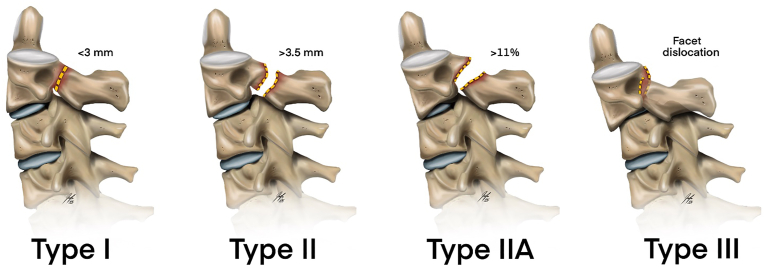
Schematic drawing of the Levine and Edwards classification for “Hangman's fractures.”

Schneider first characterized the hangman fracture in 1965 in a series of 8 cases where he observed the same fracture pattern that occurs with hangings, describing it as a bilateral fracture through the neural arch of the second cervical vertebra with or without dislocation of the body of the axis upon that of the third cervical vertebra (SCHNEIDER et al., 1965). Despite the name, later studies have noted that this trauma mechanism occurs in approximately 10% of the cases of judicial hanging (SCHNEIDER et al., 1965). The mechanism of this injury can be hyperextension-distraction, as in hanging, or hyperextension-compression, depending on the force vectors of the upper craniocervical region (Cranio-C1-C2) over C3. The first classification of these fractures was described by Effendi et al., in 1981, categorizing these fractures into three groups. Type I is an isolated hairline fracture in the axis ring, type II is a fracture with displacement of the anterior fragment and abnormal disk below the axis, and type III is a fracture with displacement of the anterior fragment and locked facet C2-C3 (Effendi et al., 1981). Although widely accepted and used, this classification factor in the degree of the displacement between C2-C3. Levine and Edwards proposed modifying the original classification in 1988, adding subgroups based on the degree of fracture displacement. In their modification, type I is a fracture through the neural arch of the axis without displacement, type II is a fracture with displacement >3 mm and significant angulation; type IIA is a fracture with displacement < 3 mm but with angulation, and type III is a fracture with severe angulation and displacement with facet dislocation (Levine and Edwards, 1985). See Fig. 3.

While this classification factors in the degree of displacement, it does not quantify angulation and fails to consider atypical fracture types. Starr and Eismont brought attention to atypical hangman fractures (AHF) in 1993, reporting from their cohort that AHF had a more significant potential for neurologic compromise (Starr and Eismont, 1993). They characterized these fractures as a posterior axis body fracture with unilateral or bilateral continuity to the posterior cortex or pedicle (Starr and Eismont, 1993).

Francis et al. published a proposed hangman's fracture classification based on a series of 123 cases, which considered the degree of displacement and angulation. Under this classification, Type I is a fracture with displacement <3.5 mm and angulation <11°, type II is a fracture with displacement <3.5 mm and angulation >11°, type III is a fracture with displacement >3.5 mm or <0.5 widths and angulation >11°, type IV is a fracture with displacement >3.5 mm or >0.5 vertebral widths and angulation >11° and Type V is a fracture with any disk disruption (Francis et al., 1981). Despite incorporating both angulation and displacement, this classification is not well known and is rarely used.

Josten and colleagues put forth a four-type classification of Hangman's fractures. They distinguished injuries affecting the anterior longitudinal ligament (ALL) as a distinct category. This is the first classification to address soft tissue injury specifically (ALL and intervertebral disc), which is recognized as a major determinant of therapeutic decision-making. In accordance with this classification system, Type I is defined as a fracture with an intact ALL and an intact disk. Type 2 is characterized by a fracture with an intact ALL and a ruptured disk. Type 3 is a fracture with ALL and disk ruptured. Finally, Type 4 is a fracture with a posteriorly dislocated and fixated facet joint (Josten, 1999).

In 2021, Goel proposed a new classification considering atlantoaxial stability in hangman fractures, which he believed to be crucial for surgical decision-making. His classification is broken into four parts, with type 1 being a fracture with no atlantoaxial or C2-C3 instability, type 2 is a fracture with the presence of C2-C3 instability and no atlantoaxial instability, type 3 is a fracture with the presence of atlantoaxial instability and no C2-C3 instability, and type 4 is a fracture with the presence of both atlantoaxial and C2-C3 instability (Goel, 2021). Most recently, in 2022, Vaccaro et al. published the new AO spine classification for upper cervical spine fractures, where C2-C3 fractures were considered type III and subdivided into type A: isolated bone injury, type B: ligament and tension band injury, and type C: displacement or translation through disc or joint. In addition, modifiers and neurological status were included (Table 3) to improve the accuracy of the treatment decision for each fracture type (Vaccaro et al., 2022b).

### 4.4 Limitations

It is important to note that this study has limitations common in any narrative review. The analysis only examined existing literature, did not include gray literature, and only included studies published in PubMed. It found that all available classification studies were based on retrospective studies, expert recommendations, and case series.

## 5 Conclusions

There are multiple classifications for C2 fractures; in many cases, there are modifications of previous classifications or expanding indications. This narrative review aimed to compile a list of these classifications so surgeons could identify the classification they wanted to follow more quickly. While no singular classification is completely all-encompassing for C2 fractures, the AO Spine Upper Cervical Fracture Classification System seems promising. It encompasses most C2 fractures while factoring in neurologic status and additional modifiers to aid clinical decision-making. Furthermore, it has been demonstrated to be highly reliable, which differentiates it from the majority of other classifications that have not been validated. Future multicenter and prospective research should be conducted to investigate the value of the different classification systems.

## Disclosure-conflict of interest

No funding was received in the creation of this manuscript, and the authors of this article have no conflicts of interest to disclose. The authors of this article did not use artificial intelligence (AI) or AI-assisted tools in the creation of this manuscript and all of them had read and approved the final version of this study.

## Declaration of competing interest

The authors declare that they have no known competing financial interests or personal relationships that could have appeared to influence the work reported in this paper.
